# Mass spectrometry imaging as a tool for evaluating the pulmonary distribution of exogenous surfactant in premature lambs

**DOI:** 10.1186/s12931-019-1144-5

**Published:** 2019-08-05

**Authors:** Riccardo Zecchi, Pietro Franceschi, Laura Tigli, Francesca Ricci, Francesca Boscaro, Barbara Pioselli, Valentina Mileo, Xabier Murgia, Federico Bianco, Fabrizio Salomone, Augusto F. Schmidt, Noah H. Hillman, Matthew W. Kemp, Alan H. Jobe

**Affiliations:** 1 Mass Spectrometry Service Center (CISM), University of Florence, Florence, Italy; 2 Computational Biology, Research and Innovation Centre, Fondazione Edmund Mach, S. Michele all’Adige, TN Italy; 3 Preclinical R&D, Chiesi Farmaceutici, Largo Francesco Belloli, 11/A, 43122 Parma, Italy; 4 Scientific Consultancy, Saarbrücken, Germany; 5 Division of Neonatology and Pulmonary Biology, Cincinnati Children’s Hospital, Cincinnati, USA; 6 Division of Neonatology, Cardinal Glennon Children’s Hospital, Saint Louis University, Saint Louis, USA; 7 Division of Obstetrics and Gynecology, University of Western Australia, Perth, WA Australia

**Keywords:** Surfactant, CHF5633, Mass spectrometry imaging, Respiratory distress syndrome, Premature lambs

## Abstract

**Background:**

The amount of surfactant deposited in the lungs and its overall pulmonary distribution determine the therapeutic outcome of surfactant replacement therapy. Most of the currently available methods to determine the intrapulmonary distribution of surfactant are time-consuming and require surfactant labelling. Our aim was to assess the potential of Mass Spectrometry Imaging (MSI) as a label-free technique to qualitatively and quantitatively evaluate the distribution of surfactant to the premature lamb.

**Methods:**

Twelve preterm lambs (gestational age 126-127d, term ~150d) were allocated in two experimental groups. Seven lambs were treated with an intratracheal bolus of the synthetic surfactant CHF5633 (200 mg/kg) and 5 lambs were managed with mechanical ventilation for 120 min, as controls. The right lung lobes of all lambs were gradually frozen while inflated to 20 cmH_2_O pressure for lung cryo-sections for MSI analysis. The intensity signals of SP-C analog and SP-B analog, the two synthetic peptides contained in the CHF5633 surfactant, were used to locate, map and quantify the intrapulmonary exogenous surfactant.

**Results:**

Surfactant treatment was associated with a significant improvement of the mean arterial oxygenation and lung compliance (*p* < 0.05). Nevertheless, the physiological response to surfactant treatment was not uniform across all animals. SP-C analog and SP-B analog were successfully imaged and quantified by means of MSI in the peripheral lungs of all surfactant-treated animals. The intensity of the signal was remarkably low in untreated lambs, corresponding to background noise. The signal intensity of SP-B analog in each surfactant-treated animal, which represents the surfactant distributed to the peripheral right lung, correlated well with the physiologic response as assessed by the area under the curves of the individual arterial partial oxygen pressure and dynamic lung compliance curves of the lambs.

**Conclusions:**

Applying MSI, we were able to detect, locate and quantify the amount of exogenous surfactant distributed to the lower right lung of surfactant-treated lambs. The distribution pattern of SP-B analog correlated well with the pulmonary physiological outcomes of the animals. MSI is a valuable label-free technique which is able to simultaneously evaluate qualitative and quantitative drug distribution in the lung.

**Electronic supplementary material:**

The online version of this article (10.1186/s12931-019-1144-5) contains supplementary material, which is available to authorized users.

## Introduction

Surfactant replacement therapy consists of delivering exogenous surfactant directly to the lungs of babies suffering from the Respiratory Distress Syndrome (RDS) [1]. Although roughly 80% of the surfactant remains within the lungs after approximately 30 min of intratracheal administration [2, 3], its intrapulmonary distribution is often non-homogenous [4, 5], which has been associated with poor clinical outcomes [6]. Pulmonary surfactant distribution is governed by a number of factors including the viscosity, surface tension and density of the surfactant preparation [7], the administration technique (e.g. instillation or aerosol delivery) [6, 8], the administration volume and injection rate [4, 9], the type of ventilation support (invasive/non-invasive), [2], the position of the patients with respect to gravity and the overall pulmonary status [10].

The investigation of the pulmonary distribution of exogenous surfactant has been the primary outcome of several investigations comparing the efficacy of different surfactant preparations [7, 10] or the feasibility of alternative surfactant administration techniques such as nebulization [2, 6]. In most studies, the pulmonary distribution of surfactant was determined with the technique of the radiolabeled microspheres [5, 6, 8, 9]. The use of this technique is unfortunately limited by the availability of appropriate facilities to handle radioactive samples. Similar techniques based on non-radioactive tracers were further developed and have also been proven useful to assess the pulmonary distribution of surfactant [11, 12]. Alternatively, imaging techniques such as gamma scintigraphy or micro-PET-CT have been employed to investigate the pulmonary distribution of surfactant [2, 3]. All these techniques rely on the use of a label which is added to the surfactant. Therefore, one must assume that the label will equally distribute with the surfactant within the lungs.

Mass-spectrometry imaging (MSI) provides complementary information to the traditional methods utilized in drug distribution studies [13] and holds great potential as a tool to investigate the intrapulmonary distribution of exogenous surfactant. MSI is particularly appealing because a wide array of both endogenous and exogenous molecules can be localized based on their mass-to-charge ratio (m/z) without the need of any sort of labeling [14, 15]. In addition, the sensitivity of MSI analysis is generally good enough to provide quantitative data for the molecule of interest [16, 17].

The aim of this work was to assess the potential of MSI analysis to simultaneously obtain qualitative and quantitative data of the pulmonary distribution of surfactant. To validate this proof of concept, we performed a MSI analysis of lung sections of preterm lambs treated with the synthetic surfactant CHF5633 (Chiesi Farmaceutici, Parma, Italy), which contains the phospholipids dipalmitoyl phosphatidylcholine (DPPC) and palmitoyloleoyl phosphatidilglycerol (POPG) in a 1:1 ratio and incorporates analogs of both hydrophobic surfactant proteins (SP): SP-B and SP-C [18, 19]. SP-B and SP-C analogs have 34 and 33 amino acid residues, respectively, the former accounting for 0.2% of the whole surfactant mass, and the latter for 1.5% of it. Our MSI strategy was based on the intrapulmonary detection, mapping and quantification of both synthetic peptides, by monitoring their characteristic molecular ions following Matrix-Assisted Laser Desorption Ionization (MALDI). Additionally, we correlated the lung distribution data from the MSI analysis with a set of physiological parameters characterizing the response of the individual animals.

## Material and methods

### Animal experiments

All animal experiments were performed after obtaining approval from the animal welfare committee of the University of Western Australia. Merino sheep were time-mated to yield lambs of 126–127 days’ gestational age (term 150 days). On the day of delivery, ewes were sedated, intubated, and mechanically ventilated. Lambs were surgically delivered. Following exposure of the fetal head, an endotracheal tube was placed surgically and the lamb was delivered. Animals were then weighed, dried, and moved to an infant warmer bed (CosyCot, Fisher&Paykel, Auckland, NZ). Mechanical ventilation was immediately started using a Fabian ventilator (Acutronic Medical Systems, Hirzel, Switzerland) and an umbilical arterial catheter was placed. Sedation/analgesia was maintained throughout the whole follow up period with intramuscular ketamine (20 mg upon initiation of ventilation, and as needed).

All lambs were initially ventilated for 15 min with the following ventilation settings: Positive Inspiratory Pressure (PIP) limited to 35 cmH_2_O, Positive End-Expiratory Pressure (PEEP) of 6 cmH_2_O, respiratory rate of 70/min, and an inspiratory time of 0.3 s. A tidal volume (V_T_) of 5 mL/kg was targeted in this initial ventilation period. The fraction of inspired oxygen (FiO_2_) was kept constant at 100%. After the initial ventilation period, lambs were allocated to one of the two experimental groups. The first group of animals was treated with a 200 mg/kg dose of CHF5633 by bolus airway instillation and further supported with mechanical ventilation for 120 min (**SF-Bolus** group, *n* = 7). One-third of the dose was delivered with the animal in the prone position, and 1/3 each in left lateral and right positions with brief ventilation between surfactant injections. A group of untreated lambs managed with mechanical ventilation for a total of 135 min (15 min of initial ventilation+ 120 min of follow-up) served as **Control** group (*n* = 5). During the follow-up period, PIP was adjusted to maintain a V_T_ of 6–7 mL/kg when possible. Arterial blood gases were assessed after the initial period of mechanical ventilation (before surfactant treatment), 5 min after surfactant treatment and then at 30 min intervals for 120 min. Heart rate (HR) and arterial blood pressure were continuously measured and recorded (SurgiVet monitors, Smiths Medical). Airway flow, mean airway pressure (MAP) and V_T_ were monitored with a flow sensor connected to the endotracheal tube. The flow sensor of the Fabian ventilator (0.9 mL dead space) was calibrated by occlusion prior to beginning of ventilation of the lambs. The lambs were sedated with ketamine, thus did not breath spontaneously and the dynamic compliance (C_dyn_) values could be reliably extracted from the ventilator. C_dyn_ was calculated by dividing the changes in lung volume (∆V, equivalent to V_T_, in mL) by the changes in pressure (∆P) standardized by the animal’s weight:

$$ {\mathrm{C}}_{\mathrm{dyn}}=\Delta  \mathrm{V}/\left(\Delta  \mathrm{P}\ast \mathrm{Weight}\right) $$

At necropsy, lungs were removed, visually inspected and separated into right and left lungs. The right lung was inflated statically with air to 35 cmH_2_O and suspended in a Dewar flask containing liquid nitrogen in the bottom. Distending pressure was then decreased to 20 cmH_2_O and the lung froze above the liquid nitrogen in the cold environment in about 15 min. The lungs were delivered frozen on dry ice to the University of Florence, where they were stored at − 80 °C until analysis.

### Sample preparation and MSI workflow

**Fig. 1:**
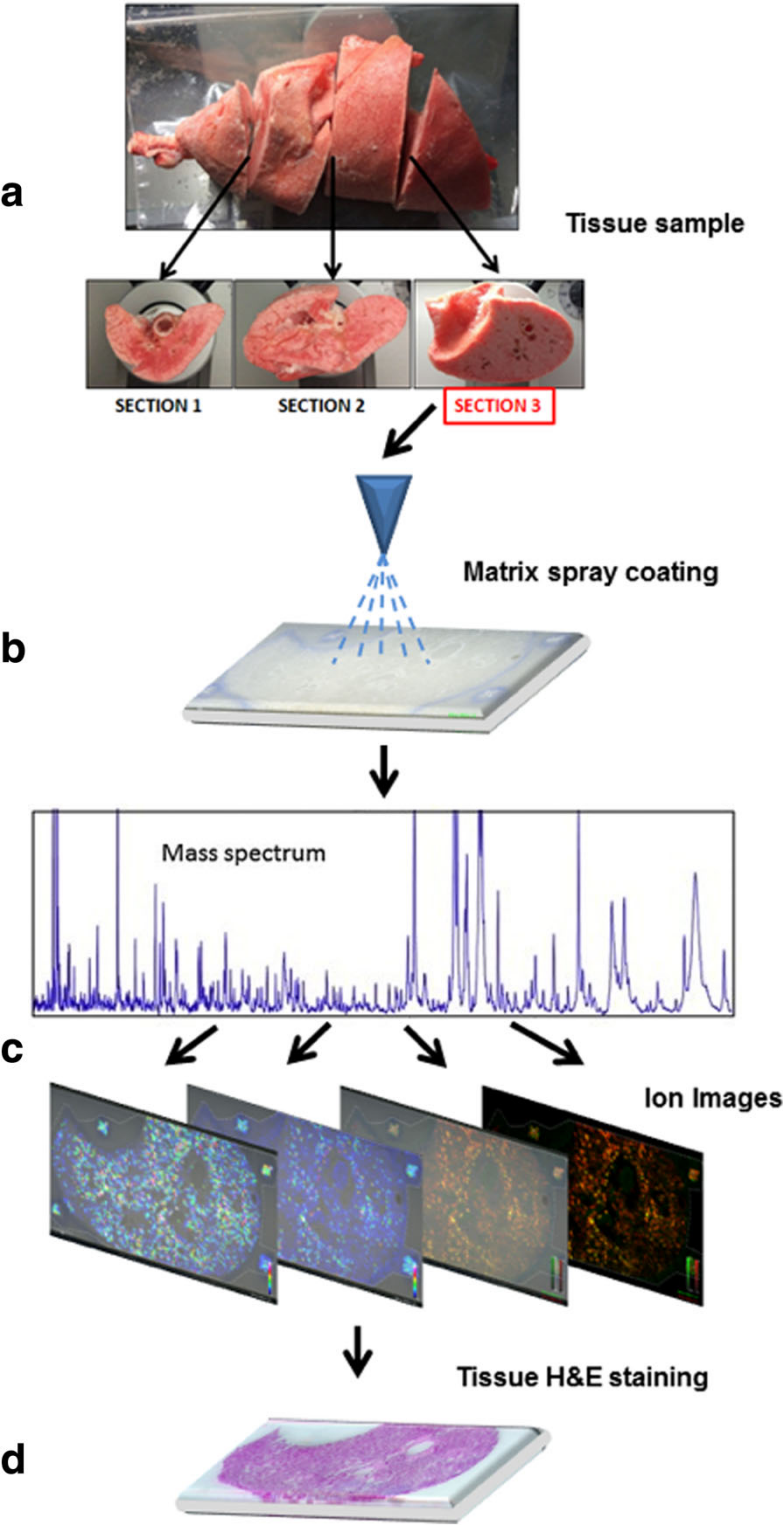
Workflow of MSI analysis. The right lungs of lambs were cut into four pieces of approximately 3 cm (**a**) and the lower face of the third piece was cryo-sectioned and thaw-mounted onto a conductive glass slide. Samples were then spray-coated with an appropriate MALDI matrix (**b**). Samples were then analyzed with MALDI-TOF/TOF instrumentation, acquiring spectra at defined spatial intervals (**c**). Lastly, data were elaborated to create 2D ion intensity maps of surfactant Protein B and C analogs and tissue slices were stained with Hematoxylin and Eosin (**d**)

Additional information of the materials and the methods describing the MSI analysis is available as supplementary information (Additional file 1: Method S1). A single premature lamb lung has an approximate size of 12 × 8 cm and its frontal section exceeds the standard size of the histology glass used for MSI analysis. Therefore, samples were transversally cut in four pieces of about the same thickness (~ 3 cm) and the lower face of the third piece was chosen for sectioning (Fig. 1a). This choice was made in order to have a fair representation of surfactant distribution in the peripheral parenchymal area.

Lung sections of 20 μm thickness were cut from surfactant-treated (*n* = 7) as well as from untreated control animals (*n* = 5) using a cryomicrotome and were thaw-mounted on conductive ITO glass and dried for 30 min in a standard desiccator.

The next steps consisted of spray-coating the lung slides (Fig. 1b) with MALDI matrix using an appropriate automated device. A 5 g/L solution of 4-chloro-α-cyanocinnamic acid in 50:50:0.2 H_2_O:ethanol: TFA solvent mixture was employed to uniformly coat the glass slides. A MALDI-TOF/TOF (Matrix-Assisted Laser Desorption Ionization and tandem Time-Of-Flight) instrument (Bruker UltraFlex III) was used for MSI data acquisition. Setting the spatial resolution at 350 μm allowed analyzing a full tissue section in approximately 8 h. The mass spectrometer worked in positive ion reflectron mode with full scan acquisition in the range 1200–4500 m/z.

Spectra were acquired by rastering the section surface with a step of 350 μm in both x and y directions. 2D ion intensity maps were then created by taking into consideration the intensities of the ions resulting from the ionization of of SP-C analog and SP-B analog (Fig. 1c-d). Among the different characteristic ions, SP-C analog distribution was monitored by its [SP-C analog+Na]^+^ adduct ion (m/z 3618), while the [SP-B analog+H]^+^ ion (m/z 3886) was selected for SP-B analog. All ion traces were extracted with a tolerance of 1 Da. All statistical analyses were performed on the log-transformed data in order to compensate for the expected non-normal distribution of MS data.

### Calibration curve preparation for quantitative MSI

A six-point calibration curve of chemical standards of SP-C analog and SP-B analog was performed by depositing drops of 0.5 μl of properly prepared solutions (50% ethanol) containing the same amount of both peptides over control homogenate lung tissue slices. Chemical standards of the SP-C analog and SP-B analog were synthesized and purified by Chiesi Farmaceutici laboratories (Parma, Italy). The absolute quantities of both peptides in the 0.5 μl drops were as follows: point #1: 1 ng of both peptides, point #2: 2.5 ng, point #3: 5 ng, point #4: 10 ng, point #5: 25 ng, point #6: 50 ng. To be able to directly identify the deposition area of the standards on the MSI datasets, 6.25 ng of melittin standard were added to the peptide mixture. A set of preliminary tests was performed to optimize the melittin concentration. After spotting, the samples were sectioned, coated with MALDI matrix and analyzed as described above. Three sample replicates for each calibration curve were prepared and included in calculations.

### Software

The individual data files were exported in the open-source imzML format by using the instrument control software [20]. The individual ion images were extracted from the imzML raw dataset by using a custom *Python* library relying on the pyimzML parser. All data analyses were performed in *R* (https://www.R-project.org/), relying on a set of packages to perform specific tasks. In particular: *Tidyverse* and *magrittr* for data handling and visualization, *EBImage* for image segmentation, *Lme4* for calibration curve estimation and *raster*, *sp* and *rgeos* for spatial statistics.

### Calibration curve optimization and estimation

Calibration curves for SP-C analog were estimated from the measured signal for the [SP-C analog+Na]^+^ adduct ion from the results of three independent experiments (2 replicates) by applying a mixed effect modeling approach. Prior to modeling, calibration spots were preprocessed as follows: **1**) In each experiment (and for each ion) the pixels were classified into two groups and the lower intensity population was used to identify the background pixels. A clustering approach on the log-transformed intensity was used to distinguish the two populations. The “average” signal of the low-intensity component was subtracted to compensate for the expected sample-to-sample analytical variability; **2**) Mellitin signal was used to identify the pixels which were actually spotted with the standard mixture; **3**) The average concentration per pixel in each dilution was calculated; and **4**) The best estimate for the calibration curve was obtained by applying a mixed effects modeling approach:

$$ SP-C\ {analog}_{pixel}=3.1\ast {c}_{pixel}+0.2 $$

where *SP-C analog*_*pixel*_ represents the log10-transformed signal of the [SP-C analog+Na]^+^ ion in each pixel and *c*_*pixel*_ is the actual spotted amount of SP-C analog in nanograms. The calibration curves showed good reproducibility. The 95% confidence intervals for the curve parameters were also calculated from the fitting procedure and were used to estimate the corresponding 95% confidence intervals on the absolute concentrations.

### MSI data normalization and preprocessing

**Fig. 2:**
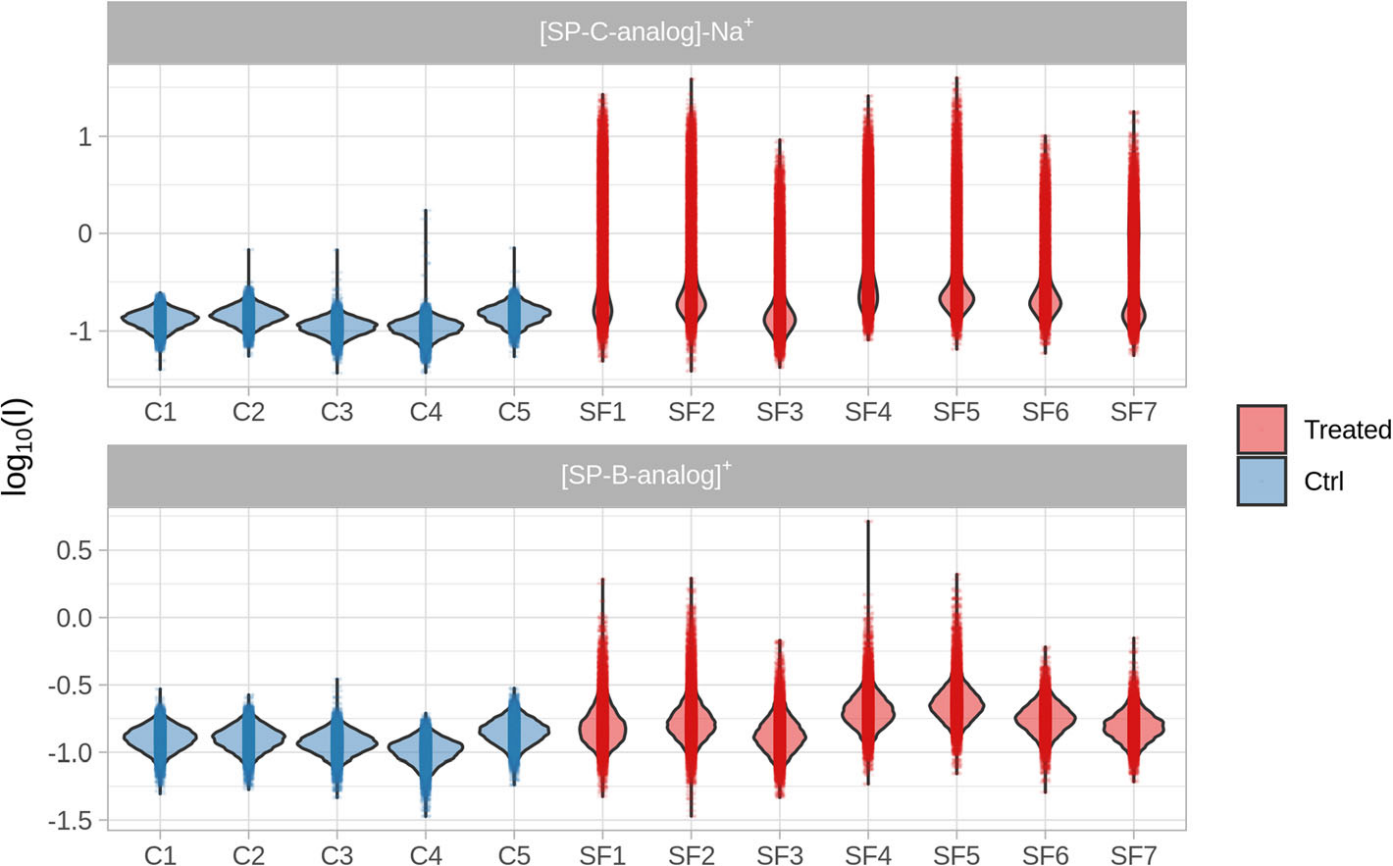
Violin plot showing the intensity distribution of SP-B analog and SP-C analog (measured as the SP-C analog-Na^+^ ion) in surfactant-treated (Treated, SF) and in untreated control (Ctrl, C) animals

The intensity measured in all pixels for all the sample is shown in Fig. 2.

**Fig. 3:**
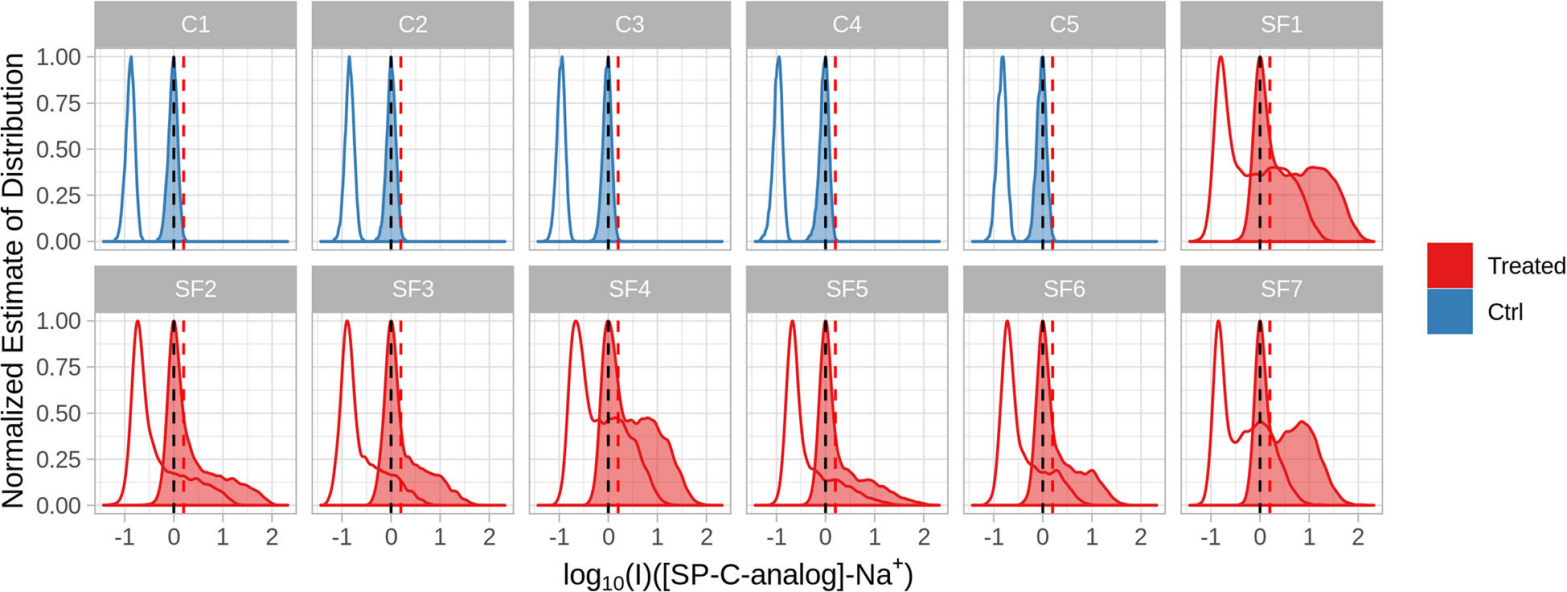
Density plot showing the effects of the batch effect removal strategy on the SP-C analog-Na^+^ ionic signal for surfactant-treated (Treated, SF) and in untreated control (Ctrl, C) animals. Empty lines show the normalized estimate of the probability distribution prior to batch correction. Vertical black lines show the mode of the distribution showing the average intensity of the background pixels after correction. Vertical red lines show the estimated threshold to identify background pixels

Each point in the plot displays the intensity of the two ions at that specific position. The “violin” envelope shows, instead, the density of the pixels along the intensity axis. The plot for SP-C analog clearly allows distinguishing treated and untreated samples. In untreated animals, however, the density profile shows a symmetric and almost normal distribution, in line to what would be expected in the presence of only background noise. In the cases of treated samples, a higher intensity component is superimposed to the “noise” distribution and is composed of the pixels where a real signal corresponding to one of the peptides was actually recorded. Considering their relative abundance in the surfactant, SP-C analog shows a stronger signal compared to SP-B analog, as expected. In addition, the variable position of the maximum of the noise distribution indicates the presence of relevant differences in response across the different samples. This type of sample-to-sample variability of analytical origin is almost unavoidable in MSI and it is, therefore, necessary to compensate in order to make the results from different experiments comparable. In this case, batch effect removal was performed by automatically subtracting to all intensities the mode of the density distribution, which in practical terms means to set the mean of the background intensity to zero. The effects of such a form of batch correction are shown in Fig. 3.

After batch correction, the density profile of untreated samples was used to estimate a hard threshold (0.20) as the 99.5 quantile of the overall intensity distribution. This threshold was then used to identify the pixels belonging to the background. The same approach was applied to the SP-B analog signal (threshold = 0.20).

In order to check the association of MSI data with the physiological parameters, semi-quantitative results for the two synthetic peptides in the different animals were summarized in terms of the log10 average intensity of the ion in the tissue section (Avg_log_10__I) and the absolute signal of the ion in the section from those pixels with a signal above the noise level (Σ_log_10__I).

### Spatial statistics

To study the dependence of the SP-C analog signal on the distance from the tissue border, the pixels belonging to each tissue section were grouped in a series of non-overlapping bins taking into account their distance from the tissue border (in the range from 0.7 and 35.5 pixels). Each bin signal was calculated as the median value of the ionic signal recorded on the individual pixels.

### Processing of physiological parameters

To compensate for an observed non-normality in the distribution of arterial oxygen partial pressure (PaO_2_) and PaCO_2_, these parameters were log10-transformed prior to analysis. Animal-specific profiles were summarized considering their area under the curve (AUC) and the value at the final time point (120 min). PaO_2_, PaCO_2_, pH, base excess (BE), C_dyn_, PIP, V_T_, MAP, HR, arterial systolic blood pressure (SBP), arterial diastolic blood pressure (DBP), mean arterial blood pressure (MABP), arterial oxygen saturation (SaO_2_), and temperature were included in the analysis.

Statistical comparisons between the physiological parameters of SF-Bolus and Control groups were performed by Student’s t-test with Levene’s test for equality of variances. The correlation between the physiological parameters and the MSI data (Avg_log_10__I and Σ_log_10__I) was investigated by correlation analysis.

## Results

### Physiological measurements

There were no significant differences between groups in terms of gestational age (Control group: 126.5 ± 0.6; SF-Inst group: 126.4 ± 0.5), body weight or fetal pH and PaCO_2_ values (Table 1). According to fetal pH and PaCO_2_ values, lambs were healthy before delivery. Nevertheless, after 15 min of mechanical ventilation, animals in both groups had severe acidosis, hypercarbia, low arterial oxygenation, and low C_dyn_ values, indicating lung immaturity.

**Table 1:** Fetal status and physiological measurements after 15 min of mechanical ventilation prior to surfactant treatment

Group	BW (kg)	*Fetal (cord blood)*		*Values after 15 min of mechanical ventilation*				
		pH	PaCO_2_ (mmHg)	pH	PaCO_2_ (mmHg)	PaO_2_ (mmHg)	PIP (cmH_2_O)	C_dyn_ (ml/kg/cmH_2_O)
Control (*n* = 5)	2.7 ± 0.5	7.26 ± 0.02	68 ± 6	7.07 ± 0.07	110 ± 25	59 ± 22	34.2 ± 2.2	0.15 ± 0.05
SF-Inst (*n* = 7)	2.7 ± 0.4	7.22 ± 0.4	75 ± 9	7.01 ± 0.15	123 ± 40	52 ± 27	34.3 ± 1.3	0.12 ± 0.05

*BW* Birth weight, *PaO*_*2*_ Arterial oxygen partial pressure, *PaCO*_*2*_ Arterial carbon dioxide partial pressure, *PIP* Peak inspiratory pressure, *C*_*dyn*_ Dynamic compliance

**Fig. 4:**
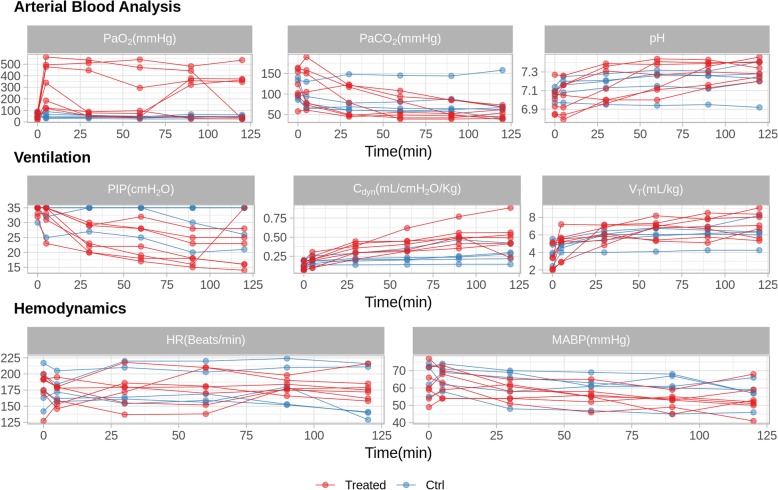
Time course of the physiological parameters for each animal included in the study. Animals treated with surfactant are represented by red lines (Treated) and untreated control animals are represented by blue lines (Ctrl). PaO_2_, arterial oxygen partial pressure; PaCO_2_, arterial carbon dioxide partial pressure; PIP, peak inspiratory pressure; C_dyn_, dynamic compliance; MAP, mean airway pressure; V_T_, tidal volume; HR, heart rate; MABP, mean arterial blood pressure

Individual time-course curves of the physiological parameters of each lamb included in the study are summarized in Fig. 4. Generally, surfactant treatment was associated with an increase of PaO_2_, pH, V_T_ and C_dyn_ and a decrease of PaCO_2_, PIP and MAP, as expected. Nevertheless, the physiological response to surfactant treatment was not uniform across all animals within the SF-Bolus group. The effect of surfactant treatment on the remaining physiological parameters was less apparent (Additional file 1: Figure S1)**.**

The AUC for each physiological parameter of each animal as well as the values at 120 min were used to perform a t-test comparing surfactant-treated to untreated control animals (Additional file 1: Table S1). From the outcome of the statistical analysis, *p* values were used to rank the most relevant physiological parameters according to their difference between treated and untreated animals. Among the parameters, PaO_2_AUC_ showed the lowest *p* value (*p* = 0.0092), followed by the parameters derived from C_dyn_ (C_dyn_AUC_ and C_dyn_120min_, *p* = 0.026 and *p* = 0.023, respectively) and by PIP__AUC_ (*p* = 0.038).

### MSI analysis

**Fig. 5:**
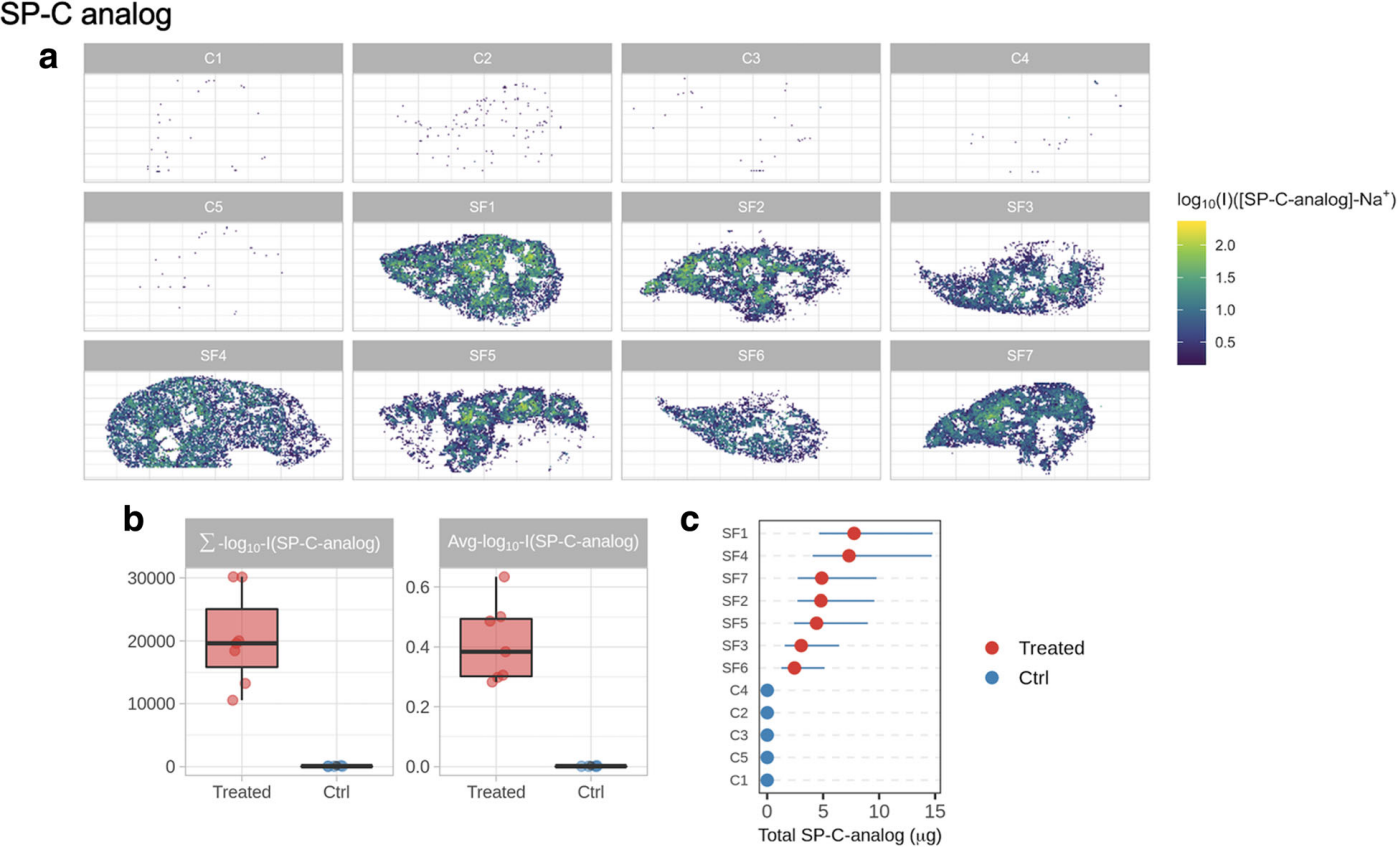
**a** Reconstructed spatial distribution images of the SP-C analog in the lungs of surfactant-treated (SF) and untreated control (C) animals. **b** Boxplots showing the difference of MSI-derived parameters (the log10 average intensity of ion inside the lung tissue section, Avg_log_10__I; and the absolute signal of the ion in the lung section from those pixels with a signal above the noise level, Σ_log_10__I) in control (Ctrl) and surfactant-treated animals. Quantification results for SP-C analog (**c**). Blue, horizontal lines in quantification charts identify the 95% confidence intervals

**Fig. 6:**
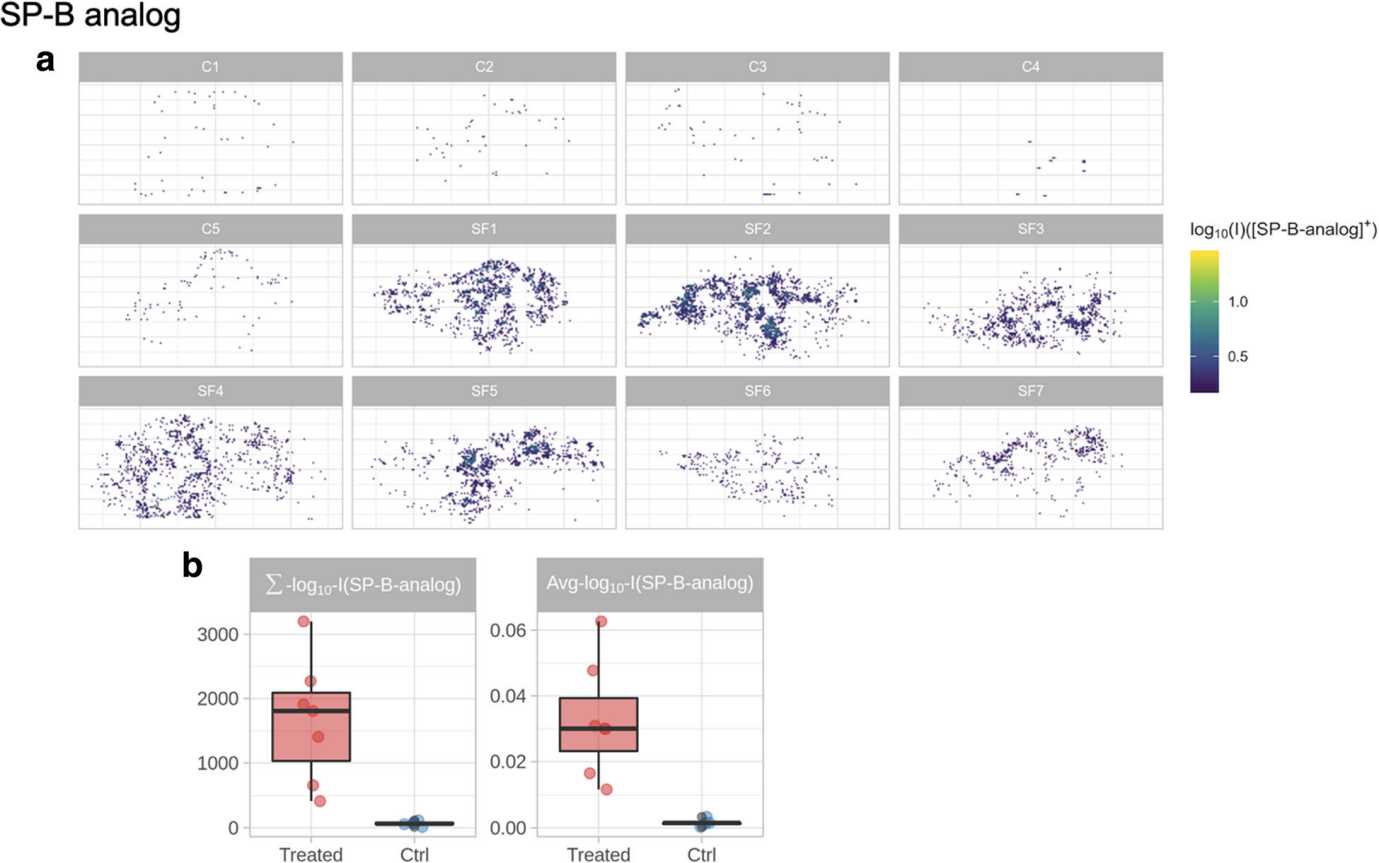
**a** Reconstructed spatial distribution images of the SP-B analog in the lungs of surfactant-treated (SF) and untreated control (C) animals. **b** Boxplots showing the difference of MSI-derived parameters (the log10 average intensity of ion inside the lung tissue section, Avg_log_10__I; and the absolute signal of the ion in the lung section from those pixels with a signal above the noise level, Σ_log_10__I) in control (Ctrl) and surfactant-treated animals

Reconstructed images based on the signals of SP-C and SP-B analogs obtained from the lower right lung of each lamb are shown in Figs. 5 and 6. Distribution maps show the presence of both peptides in all samples of surfactant-treated animals. In untreated animals, however, the intensity of the signal is remarkably low and corresponds to background noise. SP-C analog shows a fairly uniform distribution across the lung sections of surfactant-treated animals with some local accumulation of this peptide in central areas (Fig. 5a).

The distribution maps of SP-B analog show a lower intensity, mainly due to the lower abundance of this peptide compared to SP-C analog in the CHF5633 drug product but also to its relatively poor MALDI ionization yield (Fig. 6a). As for the SP-C analog, spots of local accumulation of the SP-B analog are also evident in the distribution maps. The investigation of the SP-C analog signal as a function of distance from the tissue border suggests reduced accessibility of peripheral lung regions to the drug (Additional file 1: Figure S2).

The expected difference between surfactant-treated and untreated control lungs is confirmed by the quantitative data and by the set of intensity parameters summarizing MSI results: Avg_log_10__I and Σ_log_10__I (Figs. 5 and 6b). The quantitative information on the concentration of SP-C analog is coherent among the different sections of surfactant-treated lungs, as demonstrated by the superimposing confidence intervals (Fig. 5c).

### Association of MSI data with the physiological parameters

**Fig. 7:**
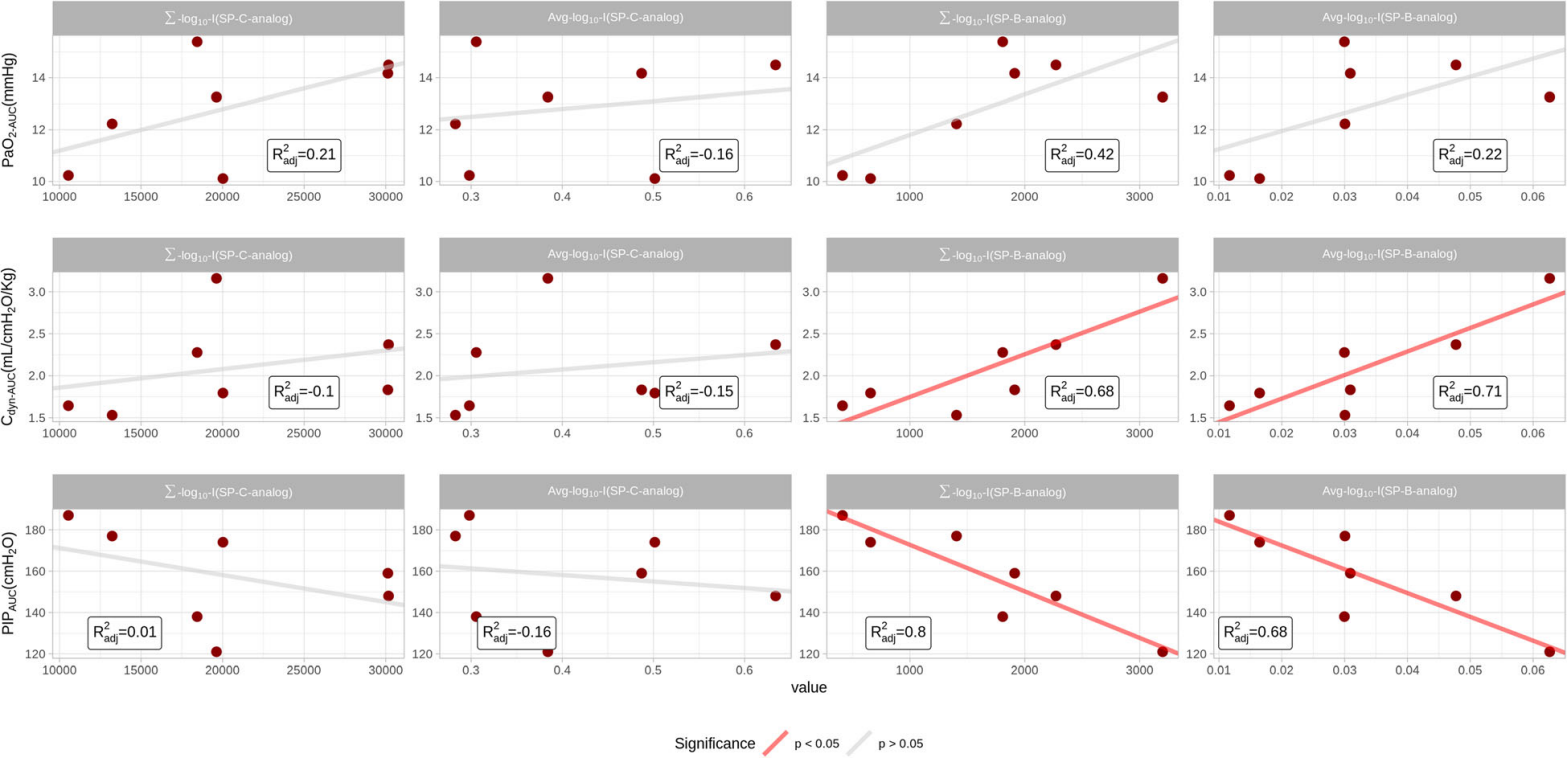
Association analysis between arterial oxygen partial pressure (PaO_2_), dynamic compliance (C_dyn_) and peak inspiratory pressure (PIP) with the log_10_ average intensity (Avg_log_10__I) and the log_10_ sum of intensity values (Σ_log_10__I) obtained for each lung section from the mass-spectrometry imaging (MSI) analysis. PaO_2_, C_dyn_ and PIP values correspond to the area under the curve (AUC) for each surfactant-treated animal

The association between the physiological parameters which were principally affected by surfactant treatment (PaO_2_AUC_, C_dyn_AUC,_ and PIP__AUC_) and the intensity parameters derived from the MSI analysis (Avg_log_10__I and Σ_log_10__I) were assessed by calculating their Spearman correlation (Fig. 7).

PaO_2_AUC_ and C_dyn_AUC_ showed a strong, positive association with the Avg_log_10__I and Σ_log_10__I of SP-B analog. PIP__AUC_ also showed a strong correlation with SP-B analog but with the opposite trend. In the case of SP-C analog, a slight association between PaO_2_AUC_, C_dyn_AUC_, and PIP__AUC_ with Σ_log_10__I persisted, while the relation between the physiological parameters and Avg_log_10__I was not apparent.

## Discussion

In this work, we applied MSI as a label-free technique to investigate the pulmonary distribution of exogenous surfactant administered to preterm lambs with RDS. By employing MSI, we were able not only to spatially resolve the location of SP-C analog and SP-B analog peptides in peripheral lung sections but also to quantify the SP-C analog concentration at spatial intervals of 350 μm. Interestingly enough, the sum of the signal intensity detected in the lower right lung for both peptides, and in particular for SP-B analog, correlated well with the pulmonary physiological parameters primarily affected by surfactant treatment, suggesting a link between the peripheral pulmonary distribution of CHF5633 and the therapeutic outcomes of surfactant-deficient lambs.

The aim of surfactant replacement therapy is to achieve an even pulmonary distribution of the surfactant in order to recruit as much collapsed alveolar areas as possible. However, early surfactant distribution studies already demonstrated a rather non-uniform pulmonary distribution following surfactant instillation [4, 5]. An even intrapulmonary surfactant distribution can be achieved in preterm animals if the surfactant is instilled at birth into the fluid-filled lung. Nevertheless, the distribution is expected to be less uniform if the surfactant is administered after a period of mechanical ventilation [21]. Surfactant distribution studies have served to compare the efficacy of different surfactant preparations, the effects of different intratracheal injections rates [9], and the outcomes after delivering surfactant by means of alterative techniques such as nebulization [6, 8]. Most of these distribution studies used radioactive microspheres premixed with the surfactant to trace the intrapulmonary fate of surfactant. Segerer et al. demonstrated that the use of radioactive tracers could be replaced by colored microspheres, which could be straightforwardly detected by spectrophotometry [9]. Since then, different non-radioactive labels have been used to address the pulmonary distribution of surfactant [11, 12]. Nonetheless, these techniques are rather laborious and require cutting the lungs in small pieces (70–200 pieces/lung pair), which must be analyzed individually [8, 10, 12]. Gamma scintigraphy and micro-PET-CT have also been used to assess surfactant distribution [2, 3]. Such imaging techniques enable real-time investigation of surfactant distribution, providing whole-lung qualitative information about the surfactant distribution and allowing for a semi-quantitative estimation of the amount of intrapulmonary surfactant in each lung region [2]. However, the use of whole-body imaging techniques during animal studies in the context of surfactant therapy is often limited by the requirement of intensive care and mechanical ventilation. This may, in turn, hamper the transfer of animals to the imaging facility and eventually the imaging process itself. Moreover, the aforementioned techniques rely on the use of tracer molecules to detect administered surfactant. Therefore, one must assume that the tracer will remain associated with surfactant for the duration of the experiment and that it will neither interact nor be degraded upon contact with the biological entities of the lungs. To address this issue, Diemel et al. covalently-labelled SP-B with a fluorescent tracer (BODIPY), which was incorporated into surfactant and administered to LPS-exposed rats [22]. The fluorescent signal of SP-B could be then detected by microscopy in histological preparations. More recently, Vukosavljevic et al. have applied linear Raman and coherent anti-Stokes Raman scattering (CARS) microscopy as a label-free tool to detect the presence of intracellular DPPC in primary human alveolar type II cells [23]. This approach, however, has been applied at the single-cell level so far.

In light of the limitations associated with the techniques applied to investigate the intrapulmonary distribution of surfactant, we envisaged MSI as a technique that could overcome a number of the aforementioned limitations, while offering significant advantages: 1) MSI provides spatially-resolved images of surfactant distribution at good resolution [13]; 2) it allows for a quantitative analysis of the analyte of interest [16, 17] (e.g. SP-C analog and SP-B analog); 3) it is based on label-free detection just requiring the ionization of the analyte of interest [14, 15]; 4) sample processing can be mostly automatized; and 5) MSI analysis does not require dedicated in vivo experiments exclusively designed to assess the pulmonary drug distribution, which enables combining MSI experiments with the assessment of other outcomes such as the physiological response to surfactant treatment (e.g. PaO_2_ or C_dyn_).

CHF5633 is a synthetic surfactant containing phospholipids and two synthetic peptides mimicking key sequences of SP-B and SP-C [18]. Native human SP-B and SP-C have 79 and 35 amino acids [24, 25], whereas SP-B analog and SP-C analog have 34 and 33 residues, respectively [18]. Therefore, differences in number and type of amino acids endow SP-B analog and SP-C analog with distinct m/z ratios, which facilitated their detection by means of MSI and allowed us to perform an accurate study of the intrapulmonary localization of CHF5633 surfactant. The analysis provided qualitative and quantitative data defining the pulmonary location as well as the concentration of the analyzed peptides. The selectivity of the technique was further validated by the absence of coherent intensity signals for SP-C analog and SP-B analog in the lungs of animals that were merely managed with mechanical ventilation and did not receive surfactant. The total amount of SP-C analog and SP-B analog in CHF5633 account for 1.5 and 0.2% of the surfactant mass, respectively [19]. The higher amount of SP-C analog in the formulation is in line with the observed 10-fold intensity difference registered after MSI analysis favoring SP-C analog. Although the distribution of both peptides throughout the lung slices of surfactant-treated animals was ample, areas of local accumulation for both peptides could be identified, suggesting a gradient-driven distribution from the central lung towards the periphery. SP-B and SP-C interact strongly with surfactant phospholipids [26]; therefore, one would expect an equivalent pulmonary distribution pattern for both analogs of the CHF5633 formulation, since it is expected that they will be transported along the airways bound to the phospholipids. MSI analysis revealed markedly different intensity profiles of both peptides across surfactant-treated animals, which indicates that the amount of surfactant reaching the peripheral right lung as well as its distribution differed from animal to animal. This animal-to-animal variability in surfactant distribution towards the lung periphery is intrinsic to bolus instillation in preterm lambs [7, 27].

A further aim of this work was to investigate if the physiological response elicited after CHF5633 administration to premature lambs would correlate with the surfactant distribution data from the MSI analysis. Previous studies have shown that the performance of CHF5633 compares positively to that of commercially available natural surfactant preparations [18, 19]. This preparation has been shown to improve the pulmonary function of premature animal models of severe RDS without alterations of hemodynamics following instillation [18, 19, 28, 29] and is currently undergoing clinical evaluation [30] (NCT02452476). In the present study, surfactant administration produced a significant improvement of oxygenation and lung compliance, as expected. Nevertheless, the level of improvement differed from animal to animal across surfactant-treated animals, which we hypothesized would correlate with the MSI study data.

In order to investigate the association between MSI and physiological data, the AUC of PaO_2_, C_dyn_ and PIP were deemed the most representative parameters of the pulmonary outcome, whereas the Avg_log_10__I and Σ_log_10__I were the selected parameters from the MSI analysis. Interestingly, the strongest correlations were found between the MSI data of SP-B analog and the PaO_2_AUC_, C_dyn_AUC_ and PIP__AUC_. This finding is in line with previous studies highlighting the crucial role of SP-B for an optimal surfactant function. Schürch et al. investigated the independent effects of SP-B and SP-C in vitro by supplementing organic lipid extracts with either one or the other peptide [31]. In this study, SP-B outperformed SP-C in terms of surface activity and stability performance, even though the simultaneous presence of both peptides in the lipid extract obtained the best performance. In vivo, the essential role of SP-B is clearly demonstrated by the death of SP-B knock-out mice shortly after birth due to respiratory failure [32], which does not occur in SP-C knock-out mice [33].

Despite the potential of MSI analysis in the preclinical development of pulmonary drugs, we would like to acknowledge some limitations of the technique evaluation in the present study. Due to the relatively large dimensions of the lungs of preterm lambs, we decided to analyze representative lung sections of the lower right lung, which we deemed appropriate to validate the proof of concept of the study and significantly reduced the MALDI-TOF/TOF scanning time. We did not collect full histological data-sets to perform a sound histological study of the samples for comparison with the MSI analyses. The localization of native ovine SP-B and SP-C was not assessed in this study due to the limited information available regarding these proteins’ sequences and post-translational modifications in the databases (e.g. UniProt). Nevertheless, there is a great interest inside the scientific community towards MSI capabilities for drug distribution studies as well as for the identification of pathological conditions in human specimens for diagnostic purposes [34]. This evidence is nowadays encouraging the instrument developers to develop new technical solutions in order to improve for example laser repetition rate to speed the analyses, laser diameter and focusing to increase the spatial resolution and new designed MALDI ion source and plates to allow the analysis of bigger tissue sections.

## Conclusion

This study provides a proof of concept for the application of MSI as an advanced analytical tool to investigate the intrapulmonary distribution of exogenous surfactant. In this work, we were able to detect, locate and quantify the amount of SP-B analog and SP-C analog in lung sections of preterm lambs treated with the synthetic surfactant CHF5633, providing lung images and quantitative data. Surfactant treatment significantly improved the arterial oxygenation and the lung compliance of preterm lambs with RDS. Interestingly, such pulmonary improvement correlated well with the distribution of the SP-B analog towards the lower right lung. This study postulates MSI as a valuable, label-free technique which is able to simultaneously obtain qualitative and quantitative data from preclinical drug distribution studies in the context of pulmonary drug delivery.

## Additional file

Additional file 1: Suplementary material and methods 1. **Figure S1.** Time course of the physiological parameters for each animal included in the study. **Table S1.** Outcome of the *t*-test comparing several physiological parameters between surfactant-treated and untreated control animals. **Figure S2.** The results of the spatial analysis of the MSI datasets measured on each surfactant-treated animal are summarized in the figure. (DOCX 529 kb)

## Data Availability

The datasets used and/or analysed during the current study are available from the corresponding author on reasonable request.

## Abbreviations

AUC: Area Under the Curve
Avg_log_10__I: Log_10_ Average Intensity of Ion in the Tissue Section
BE: Arterial Base Exccess
C_dyn_: Dynamic Compliance
DBP: Diastolic Blood Pressure
DPPC: Dipalmitoyl Phosphatidylcholine
FiO_2_: Fraction of Inspired Oxygen
HR: Heart Rate
m/z: Mass-to-Charge Ratio
MABP: Mean Arterial Blood Pressure
MALDI: Matrix Assisted Laser Desorption Ionization
MALDI-TOF/TOF: MALDI and Tandem Time-Of-Flight
MAP: Mean Airway Pressure
microPET-CT: Micro-Positron Emission Tomography–Computed Tomography
MSI: Mass Spectrometry Imaging
PaCO_2_: Arterial Carbon Dioxide Partial Pressure
PaO_2_: Arterial Oxygen Partial Pressure
PEEP: Positive End-Expiratory Pressure
PIP: Peak Inspiratory Pressure
POPG: Palmitoyloleoyl Phosphatidilglycerol
RDS: Respiratory Distress Syndrome
SaO_2_: Arterial Oxygen Saturation
SBP: Systolic Blood Pressure
SP: Surfactant Protein
SP-B: Surfactant Protein B
SP-C: Surfactant Protein C
V_T_: Tidal Volume
ΔP: Change in Pressure
ΔV: Change in Volume
Σ_log_10__I: Log_10_ Absolute Signal of the Ion in the Tissue Section

## References

[1] Speer CP, Robertson B, Curstedt T, Halliday HL, Compagnone D, Gefeller O (1992). Randomized European multicenter trial of surfactant replacement therapy for severe neonatal respiratory distress syndrome: single versus multiple doses of curosurf. Pediatrics..

[2] Linner R, Perez-de-Sa V, Cunha-Goncalves D (2015). Lung deposition of nebulized surfactant in newborn piglets. Neonatology..

[3] Salaets T, Gie A, Jimenez J, Aertgeerts M, Gheysens O, Vande Velde G (2019). Local pulmonary drug delivery in the preterm rabbit: feasibility and efficacy of daily intratracheal injections. Am J Physiol Lung Cell Mol Physiol.

[4] Van Der Bleek J, Plötz FB, Van Overbeek FM, Heikamp A, Beekhuis H, Wildevuur CRH (1993). Distribution of exogenous surfactant in rabbits with severe respiratory failure: the effect of volume. Pediatr Res.

[5] Ueda T, Ikegami M, Rider ED, Jobe AH (1994). Distribution of surfactant and ventilation in surfactant-treated preterm lambs. J Appl Physiol.

[6] Dijk PH, Heikamp A, Bambang Oetomo S (1997). Surfactant nebulisation: lung function, surfactant distribution and pulmonary blood flow distribution in lung lavaged rabbits. Intensive Care Med.

[7] Mazela J, Merritt TA, Terry MH, Gregory TJ, Blood AB (2012). Comparison of poractant alfa and lyophilized lucinactant in a preterm lamb model of acute respiratory distress. Pediatr Res.

[8] Henry MD, Rebello CM, Ikegami M, Jobe AH, Langenback EG, Davis JM (1996). Ultrasonic nebulized in comparison with instilled surfactant treatment of preterm lambs. Am J Respir Crit Care Med.

[9] Segerer H, van Gelder W, Angenent FWM, van Woerkens LJPM, Curstedt T, Obladen M (1993). Pulmonary distribution and efficacy of exogenous surfactant in lung-lavaged rabbits are influenced by the instillation technique. Pediatr Res.

[10] Terry MH, Merritt TA, Harding B, Schroeder H, Merrill-Henry J, Mazela J (2010). Pulmonary distribution of lucinactant and poractant alfa and their peridosing hemodynamic effects in a preterm lamb model of respiratory distress syndrome. Pediatr Res.

[11] Rahmel DK, Pohlmann G, Iwatschenko P, Volland J, Liebisch S, Kock H (2012). The non-intubated, spontaneously breathing, continuous positive airway pressure (CPAP) ventilated pre-term lamb: a unique animal model. Reprod Toxicol.

[12] Rey-Santano C, Alvarez-Diaz FJ, Mielgo V, Murgia X, Lafuente H, Ruiz-Del-Yerro E (2011). Bronchoalveolar lavage versus bolus administration of lucinactant, a synthetic surfactant in meconium aspiration in newborn lambs. Pediatr Pulmonol.

[13] Caprioli RM, Farmer TB, Gile J (1997). Molecular imaging of biological samples: localization of peptides and proteins using MALDI-TOF MS. Anal Chem.

[14] Pierson J, Norris JL, Aerni H-R, Svenningsson P, Caprioli RM, Andrén PE (2004). Molecular profiling of experimental Parkinson’s disease: direct analysis of peptides and proteins on brain tissue sections by MALDI mass spectrometry. J Proteome Res.

[15] Sköld K, Svensson M, Nilsson A, Zhang X, Nydahl K, Caprioli RM (2006). Decreased striatal levels of PEP-19 following MPTP lesion in the mouse. J Proteome Res.

[16] Pirman D (2015). Quantitative profiling of tissue drug distribution by MS imaging. Bioanal Futur Sci.

[17] Reyzer ML, Caprioli RM (2007). MALDI-MS-based imaging of small molecules and proteins in tissues. Curr Opin Chem Biol.

[18] Sato A, Ikegami M (2012). SP-B and SP-C containing new synthetic surfactant for treatment of extremely immature lamb lung. PLoS One.

[19] Ricci F, Murgia X, Razzetti R, Pelizzi N, Salomone F (2017). In vitro and in vivo comparison between poractant alfa and the new generation synthetic surfactant CHF5633. Pediatr Res.

[20] Schramm T, Hester A, Klinkert I, Both J-P, Heeren RMA, Brunelle A (2012). imzML — a common data format for the flexible exchange and processing of mass spectrometry imaging data. J Proteome.

[21] Jobe A, Ikegami M, Jacobs H, Jones S (1984). Surfactant and pulmonary blood flow distributions following treatment of premature lambs with natural surfactant. J Clin Invest.

[22] Diemel RV, Walch M, Haagsman HP, Putz G (2002). In vitro and in vivo intrapulmonary distribution of fluorescently labeled surfactant. Crit Care Med.

[23] Vukosavljevic Branko, Hittinger Marius, Hachmeister Henning, Pilger Christian, Murgia Xabier, Gepp Michael M., Gentile Luca, Huwer Hanno, Schneider‐Daum Nicole, Huser Thomas, Lehr Claus‐Michael, Windbergs Maike (2019). Vibrational spectroscopic imaging and live cell video microscopy for studying differentiation of primary human alveolar epithelial cells. Journal of Biophotonics.

[24] Conkright JJ, Glasser SW, Weaver TE, Na C-L, Bridges JP, Voorhout WF (2002). Secretion of surfactant protein C, an integral membrane protein, requires the N-terminal propeptide. J Biol Chem.

[25] Curstedt T, Johansson J, Barros-Söderling J, Robertson B, NilssoN G, Westberg M (1988). Low-molecular-mass surfactant protein type 1: the primary structure of a hydrophobic 8-kDa polypeptide with eight half-cystine residues. Eur J Biochem.

[26] Creuwels LAJM, Van Golde LMG, Haagsman HP (1997). The pulmonary surfactant system: biochemical and clinical aspects. Lung..

[27] Rey-Santano C, Mielgo VE, Andres L, Ruiz-del-Yerro E, Valls-i-Soler A, Murgia X (2013). Acute and sustained effects of aerosolized vs. bolus surfactant therapy in premature lambs with respiratory distress syndrome. Pediatr Res.

[28] Rey-Santano C, Mielgo VE, Murgia X, Gomez-Solaetxe MA, Salomone F, Bianco F (2017). Cerebral and lung effects of a new generation synthetic surfactant with SP-B and SP-C analogs in preterm lambs. Pediatr Pulmonol.

[29] Seehase M, Collins JJP, Kuypers E, Jellema RK, Ophelders DRMG, Ospina OL (2012). New surfactant with SP-B and C analogs gives survival benefit after inactivation in preterm lambs. PLoS One.

[30] Sweet DG, Turner MA, Straňák Z, Plavka R, Clarke P, Stenson BJ (2017). A first-in-human clinical study of a new SP-B and SP-C enriched synthetic surfactant (CHF5633) in preterm babies with respiratory distress syndrome. Arch Dis Child Fetal Neonatal Ed.

[31] Schürch D, Ospina OL, Cruz A, Pérez-Gil J (2017). Combined and independent action of proteins SP-B and SP-C in the surface behavior and mechanical stability of pulmonary surfactant films. Biophys J.

[32] Clark JC, Wert SE, Bachurski CJ, Stahlman MT, Stripp BR, Weaver TE (1995). Targeted disruption of the surfactant protein B gene disrupts surfactant homeostasis, causing respiratory failure in newborn mice. Proc Natl Acad Sci.

[33] Glasser SW, Sly PD, Ross GF, Ikegami M, Korfhagen TR, Na C-L (2002). Altered stability of pulmonary surfactant in SP-C-deficient mice. Proc Natl Acad Sci.

[34] Ouyang Y, Liu J, Nie B, Dong N, Chen X, Chen L (2017). Differential diagnosis of human lung tumors using surface desorption atmospheric pressure chemical ionization imaging mass spectrometry. RSC Adv.

