# Supplementary material for: Mass spectrometry imaging as a tool for evaluating the pulmonary distribution of exogenous surfactant in premature lambs
Source: Respir Res. 2019 Aug 5;20:175. doi: 10.1186/s12931-019-1144-5 (PMC6683365; doi:10.1186/s12931-019-1144-5)

Additional file 1

MASS SPECTROMETRY IMAGING AS A TOOL FOR EVALUATING THE PULMONARY DISTRIBUTION OF EXOGENOUS SURFACTANT IN PREMATURE LAMBS

Riccardo Zecchi^1#^, Pietro Franceschi^2#^, Laura Tigli^3^, Francesca Ricci^3^, Francesca Boscaro^1^ Barbara Pioselli^3^, Valentina Mileo^3^, Xabier Murgia^4^, Federico Bianco^3^, Fabrizio Salomone^3*^, Augusto F Schmidt^5^, Noah H Hillman^6^, Matthew W Kemp^7^, Alan H Jobe^6,7^

^1^ Mass Spectrometry Service Center (CISM), University of Florence, Florence, Italy

^2^ Computational Biology, Research and Innovation Centre, Fondazione Edmund Mach, S. Michele all'Adige (TN), Italy

^3^ Preclinical R&D, Chiesi Farmaceutici, Parma, Italy

^4^ Scientific Consultancy, Saarbrücken, Germany

^5^ Division of Neonatology and Pulmonary Biology, Cincinnati Children’s Hospital, Cincinnati, USA

^6^ Division of Neonatology, Cardinal Glennon Children’s Hospital, Saint Louis University, Saint Louis, USA

^7^ Division of Obstetrics and Gynecology, University of Western Australia, Perth, WA, Australia

^*^Corresponding Author: Fabrizio Salomone, PhD. Largo Francesco Belloli, 11/A 43122, Parma, ITALY; Tel. +39 0521 1689158. E-mail: f.salomone@chiesi.com

^#^these authors contributed equally

**SUPLEMENTARY MATERIAL AND METHODS 1**

*Materials for MSI Analysis*

Analytical grade solvents (H_2_O, ethanol and xylene) as well as MALDI matrix (4-chloro-α-cyanocinnamic acid), trifluoroacetic acid and melittin standard peptide were purchased from Sigma Aldrich (Milan, Italy). Meyer hematoxylin, eosin G and Micromount medium were purchased from Diapath (Bergamo, Italy). ITO glass slides were purchased from Bruker Daltonics (Bremen, Germany). Chemical standards of the two peptides present in the drug formulation (CHF4902.03 named as SP-C analog and CHF5736.03 named as SP-B analog) were synthetized and purified by Chiesi Farmaceutici laboratories (Parma, Italy).

*Spatial Statistics*

To study the dependence of the SP-C analog signal on the distance from the tissue border, the pixels belonging to each tissue section were grouped in a series of non-overlapping bins taking into account their distance from the tissue border (in the range from 0.7 and 35.5 pixels). Each bin signal was calculated as the median value of the ionic signal recorded on the individual pixels.

**Figure S1**

Time course of the physiological parameters for each animal included in the study. Animals treated with surfactant are represented by red lines (Treated) and untreated control animals are represented by blue lines (Ctrl).

**
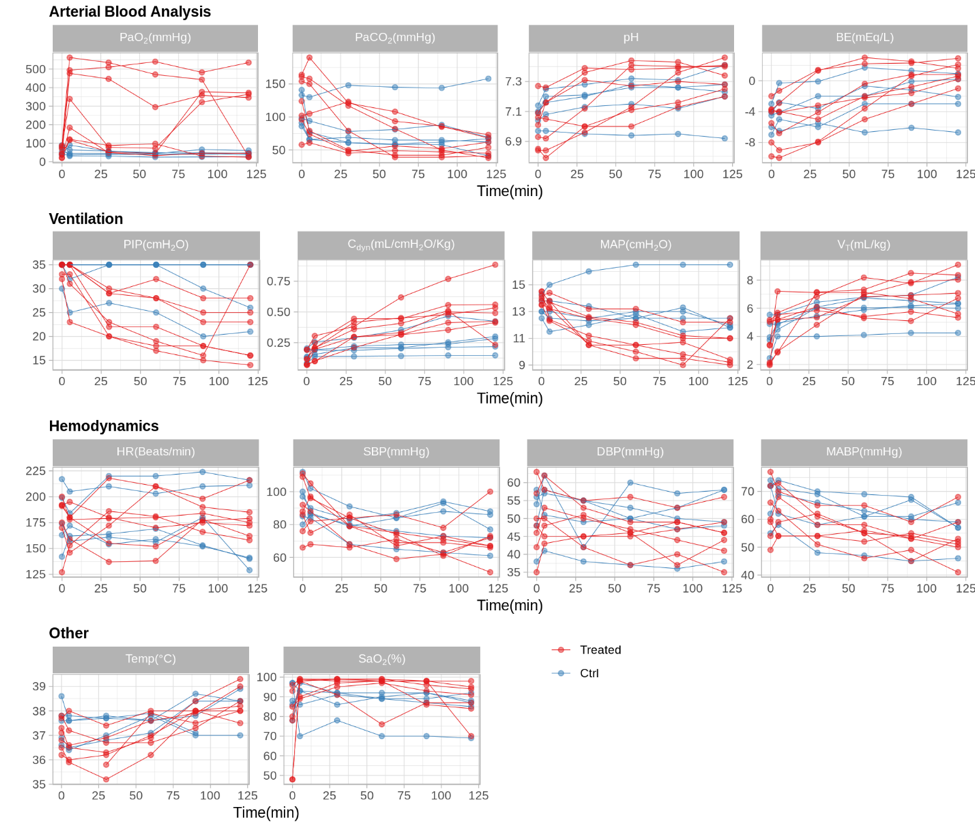
**

PaO_2_, arterial oxygen partial pressure; PaCO_2_, arterial carbon dioxide partial pressure; BE, base excess; PIP, peak inspiratory pressure; C_dyn_, dynamic compliance; MAP, mean airway pressure; V_T_, tidal volume; HR, heart rate; SBP, arterial systolic blood pressure; DBP, arterial diastolic blood pressure; MABP, mean arterial blood pressure; Temp, Temperature; SaO_2_, arterial oxygen saturation.

**Table S1**

Outcome of the *t*-test comparing several physiological parameters between surfactant-treated and untreated control animals. The table displays the *t*-scores and *p*-values for each parameter. ****

AUC, area under the curve; BE, arterial base excess; Cdyn, dynamic compliance; DBP, diastolic blood pressure; HR, heart rate; CO_2_, arterial partial pressure of carbon dioxide; O_2_, arterial partial pressure of oxygen; MAP, mean airway pressure; PIP, peak inspiratory pressure; Sat, oxygen saturation; SBP, systolic arterial pressure; temp, temperature; Vt/kg, tidal volume.

**Figure S2**

The results of the spatial analysis of the MSI datasets measured on each surfactant-treated animal are summarized in the figure. The plot displays the relation between the SPC-analog-Na^+^ signal and the distance from the tissue border. The presence of a common increasing trend is clear; suggesting a reduced accessibility of surfactant towards the peripheral lung regions.


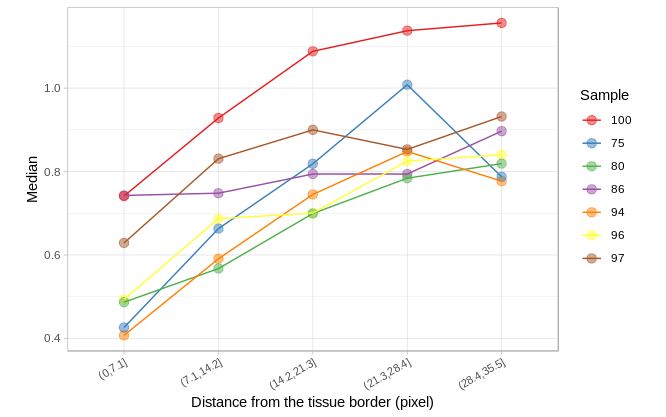

Supplement: Supplementary file 1 — Suplementary material and methods 1. Figure S1. Time course of the physiological parameters for each animal included in the study. Table S1. Outcome of the t-test comparing several physiological parameters between surfactant-treated and untreated control animals. Figure S2. The results of the spatial analysis of the MSI datasets measured on each surfactant-treated animal are summarized in the figure. (DOCX 529 kb) [file 12931_2019_1144_MOESM1_ESM.docx]
